# NLGenomeSweeper: A Tool for Genome-Wide NBS-LRR Resistance Gene Identification

**DOI:** 10.3390/genes11030333

**Published:** 2020-03-20

**Authors:** Nicholas Toda, Camille Rustenholz, Agnès Baud, Marie-Christine Le Paslier, Joelle Amselem, Didier Merdinoglu, Patricia Faivre-Rampant

**Affiliations:** 1 Université Paris-Saclay, INRAE, Etude du Polymorphisme des Génomes Végétaux (EPGV), 91000 Evry, France; ntoda03@gmail.com (N.T.); marie-christine.le-paslier@inrae.fr (M.-C.L.P.); 2 Université de Strasbourg, INRAE, SVQV UMR A 1131, 68000 Colmar, France; camille.rustenholz@inrae.fr (C.R.); didier.merdinoglu@inrae.fr (D.M.); 3 Université Paris-Saclay, INRAE, URGI, 78026 Versailles, France; agnesbaud@gmail.com (A.B.); joelle.amselem@inrae.fr (J.A.)

**Keywords:** NLR disease resistance genes, NLR-Parser, functional annotation

## Abstract

Although there are a number of bioinformatic tools to identify plant nucleotide-binding leucine-rich repeat (NLR) disease resistance genes based on conserved protein sequences, only a few of these tools have attempted to identify disease resistance genes that have not been annotated in the genome. The overall goal of the NLGenomeSweeper pipeline is to annotate NLR disease resistance genes, including RPW8, in the genome assembly with high specificity and a focus on complete functional genes. This is based on the identification of the complete NB-ARC domain, the most conserved domain of NLR genes, using the BLAST suite. In this way, the tool has a high specificity for complete genes and relatively intact pseudogenes. The tool returns all candidate NLR gene locations as well as InterProScan ORF and domain annotations for manual curation of the gene structure.

## 1. Introduction

Plants have evolved a complex system to defend against diseases and pests. A general first response involves recognition of conserved microbial or pathogen features by receptors at the surface of the cell, but pathogens may be able to shut down this general response through the production of effector proteins that inhibit the immune response and make the plant susceptible again [1]. These effectors may be recognized by a second immune response step involving resistance (R) genes that trigger effector-triggered immunity (ETI), which often leads to cell death [1]. 

Plant R genes are most commonly nucleotide-binding leucine-rich repeat genes, referenced hereafter as NBS-LRRs. NBS-LRRs share a similar NB-ARC domain and variable C-terminal leucine-rich repeat (LRR) domains that provide target specificity [2]. The NB-ARC domain is the most conserved region of NBS-LRR genes [3,4]. Different classes are characterized by their N-terminal domain [5]. Genes without the following N-terminal domains are classified as NLs: N—NB-ARC; L—LRRs. CNLs contain an N-terminal coiled-coil domain, TNLs contain an N-terminal Toll-interleukin receptor-like (TIR) domain, and RNLs contain an N-terminal RPW8 domain. 

To date, NBS-LRRs have been difficult to predict using automatic gene annotation tools, due to their duplicated and clustered nature leading to fragmented or absent annotations [2,5,6,7]. This is compounded by the fact that NBS-LRRs are sometimes annotated as being repetitive sequences and so are present in repeat databases [8]. Additionally, some disease resistance genes are lowly expressed except during infection so RNA-Seq data would not provide supporting evidence for gene annotation. Pipelines to identify NBS-LRRs genes have traditionally attempted to identify conserved domains in protein sequences based on gene annotations [2,9,10,11]. A few tools, notably NLR-Annotator (an expanded version of NLR-Parser), have also attempted to identify unannotated disease resistance genes from whole genome sequences by searching for NBS-LRR-related motifs in large nucleotide sequences [12]. Advances in next generation and sequencing technologies have led to a large expansion in the number of whole genomes available, and now third generation sequencing technologies are allowing for a more accurate assembly of genomic regions containing repeated and clustered NBS-LRR genes. It is, therefore, increasingly important to correctly identify NBS-LRR genes in these regions, which often proves difficult for automatic gene annotation. 

Here, we present NLGenomeSweeper, a pipeline dedicated to the annotation of NBS-LRR disease resistance genes in genome assemblies. It is based on the identification of a complete NB-ARC domain and the creation of class-specific NB-ARC domain consensus sequences. The outputs of this pipeline are candidate locations with additional functional domain information predicted by InterProScan. The output BED and GFF files are designed to be used as inputs of the genome browser for expert manual annotation of NBS-LRR genes. 

## 2. Materials and Methods

### NLR Candidate Identification Pipeline

The pipeline uses a double pass process for NBS-LRR gene identification in the genome, based on methods used in previous manual genome-wide screens using BLAST to search the genome for the NB-ARC domain [5,13]. The first pass focuses on the identification of initial NBS-LRR candidates using the NB-ARC domain, as it is the most conserved region present in all NBS-LRR genes. During the first pass, tBLASTn [14] is used to search the genome, using the NB-ARC sequences based on the Pfam profile (PF00931) [15] and custom consensus sequences based on the four classes of *Vitis vinifera* NBS-LRR genes (CNL, TNL, NL, and RNL) retrieved from NCBI. Overlapping hits are merged and then adjacent hits on the same strand are combined (within 1000 bp) to handle small regions of divergence and introns. Hits must have a length greater than 80% of the most similar NB-ARC sequence in order to be retained as candidates. The sequences obtained in this step are used to build a species-specific HMM profile: they are translated into peptides using TransDecoder [16], submitted to multiple alignment with MUSCLE [17], and used to create custom NB-ARC sequences with HMMER (hmmer.org). A second pass of NBS-LRR candidate identification is carried out using these new species and class-specific consensus sequences. Candidate *loci*, as well as 10 kb of flanking sequence on both sides, are submitted to InterProScan [18] (using the programs Coils, Gene3D, SMART and Pfam) to identify domains and ORFs based on the nucleotide sequence. Candidates are removed if they do not contain a LRR in their flanking region. The candidate *loci* (indicating the NB-ARC domain) and InterProScan results are exported in BED and GFF3 formats, respectively, for importing into a genome browser for manual annotation. Pseudogenes with a complete NB-ARC domain will also be identified by the pipeline, but the results are meant to be used with expert manual annotation and so pseudogenes should be dealt with at that step. The NLGenomeSweeper pipeline and additional usage information is available on GitHub (https://doi.org/10.15454/DS6VIK).

## 3. Results

### 3.1. Validation on Existing Datasets

For validation, the pipeline was run on *Arabidopsis thaliana* and *Helianthus annuus* genomes. NLR-Annotator was also run for comparison, if results from this had not been previously reported. 

#### 3.1.1. *Arabidopsis thaliana*

The TAIR 10.1 genome sequence and its annotation were downloaded from the NCBI FTP repository. The *A. thaliana* NBS-LRR genes provide a good measure of the performance of the pipeline, as this is a high-quality manual annotation. A total of 146 previously identified NBS-LRR genes were retrieved [5]. NLGenomeSweeper identified 152 candidates, including 140 of the 146 previously identified NBS-LRRs (Supplementary 1). This represents a 96% sensitivity with the true negative rate comprising the rest of the genome. This set of 152 predicted genes includes the two RNL genes (AT1G33560 and AT5G66900) not identified by NLR-Annotator [12]. The authors of NLR-Parser (based on NLR-Annotator) previously noted that RNL genes are difficult to identify on the basis of the consensus motifs used [10]. When looking at the six false negatives (out of 146) not retrieved by the pipeline, we noticed that one had an intron in the NB-ARC larger than 1 kb (the threshold based on the distribution of intron sizes for all genes, although this parameter can be changed by the user), two had a NB-ARC domain shorter than the length cutoff used to exclude truncated domains, and three NBS-LRR genes were not detected using BLAST and represent a limitation to the method. A total of 12 additional candidates were identified. Six of these candidates correspond to complete CNL or TNL genes that have been added to the annotation since the original identification of NBS-LRRs. The 6 false positives are partial gene fragments (CN and TN) with full-length NB-ARC domains and are pseudogenes expected to be identified by the pipeline.

#### 3.1.2. *Helianthus annuus*

The *H. annuus* genome and annotation version 1.2 were downloaded from Phytozome [19]. The results were compared to information provided by the published automatic annotation of NBS-LRR genes [20]. This provides a complementary view on performance, as the genome was automatically annotated and NBS-LRR genes were identified based on protein sequences. The results of the pipeline and NLR-Annotator were compared to information provided by the published automatic annotation of NBS-LRR genes (Supplementary 2). Among the 352 NBS-LRR genes previously identified, only 293 had both a NB-ARC domain and LRRs. NLGenomeSweeper and NLR-Annotator identified 503 and 603 candidates, respectively. Looking at the genes missed by NLGenomeSweeper, we noticed that 80.7% contained two or fewer domains, suggesting that they are gene fragments; we do not know if this is due to structural variation or misassembly of the genomic region. The remaining genes (19.3%) had either a truncated NB-ARC domain or introns larger than 1 kb (in one case). In comparison, only 43.5% of the genes missed by NLR-Annotator contained two or fewer domains. In particular, NLR-Annotator only identified 2 out of the 10 RNL genes, where NLGenomeSweeper identified 8.

## 4. Discussion

Identification of NBS-LRR genes is an important step in the understanding of plant disease resistance. The most important step of the identification of NBS-LRR genes has in the past relied on gene predictions, which may not be accurate or exhaustive. NLGenomeSweeper allows for an accurate automated identification of NBS-LRR candidates without the requirement for previous gene predictions or additional functional information and shows broader performance than a previous tool, NLR-Annotator, which has poor performance for RNL genes. Moreover, the output format is designed to support downstream manual annotation by providing information on surrounding ORFs (Open Reading Frame) and potential functional domains. This candidate identification and supporting information becomes increasingly important as the ease of generating high-quality genome assemblies from long-read data increases and the necessity of identifying disease resistance genes genome-wide becomes more common. This pipeline will be used to expand efforts to analyze NBS-LRRs in diverse plant species.

## Supplementary Materials

The following are available online at https://www.mdpi.com/2073-4425/11/3/333/s1, Supplementary Table S1: NBS-LRR genes in *Arabidopsis thaliana*; Supplementary Table S2: NBS-LRR genes in *Helianthus annuus*.
